# Primary Cardiac Burkitt Lymphoma Presenting with Abdominal Pain

**DOI:** 10.1155/2014/687598

**Published:** 2014-11-06

**Authors:** Dimitrios Tzachanis, Rajan Dewar, Katarina Luptakova, James D. Chang, Robin M. Joyce

**Affiliations:** ^1^Cedars-Sinai Medical Center, 8700 Beverly Boulevard, AC1046-A, Los Angeles, CA 90048, USA; ^2^Beth Israel Deaconess Medical Center, 330 Brookline Avenue, Boston, MA 02215, USA

## Abstract

We describe the case of a 44-year-old woman with primary Burkitt lymphoma of the heart who presented with abdominal bloating and epigastric discomfort secondary to tamponade physiology caused by a large pericardial effusion. The pericardial fluid contained a large number of highly atypical lymphocytes with moderate basophilic cytoplasm, rare punched-out vacuoles, a vesicular nuclear chromatin, large nucleolus, and marginated chromatin that by FISH were positive for the 8;14 translocation. She had no other sites of disease. She was treated with four alternating cycles of modified CODOX-M and IVAC in combination with rituximab and remains in remission more than 5 years since diagnosis.

## 1. Background

Primary cardiac tumors are rare. Most of them are benign with myxoma being the most frequent histologic type [1–3]. Primary malignant cardiac tumors are 100–1000 times less common than metastatic disease to the heart. The most frequent primary malignant cardiac cancer is sarcoma. Lymphomas are diagnosed in about 1-2% of patients with a primary cardiac tumor and occur more frequently in immunosuppressed hosts such as in patients infected with the human immunodeficiency virus (HIV) [4]. Lymphomas are the second most common tumors involving the heart in HIV infected patients reflecting the higher incidence of lymphoma and of extranodal involvement in patients with HIV [5].

Diffuse large B cell lymphoma is the most common histologic type of cardiac lymphoma. Primary cardiac Burkitt lymphoma (BL) has been reported very rarely. In our review of the literature we identified 4 cases in adults. Kuroda et al. reported the case of a 79-year-old female with cardiac BL in the form of a left ventricular mass and a massive pericardial effusion. Diagnosis was made by pericardiocentesis that showed large B cells that had an t(8;22) translocation. The patient initially went into remission with chemotherapy with CVP (cyclophosphamide, vincristine, and prednisone) but relapsed in the brain and eventually succumbed to her disease [6]. Fatimi et al. reported the case of an elderly man with primary cardiac BL who was initially misdiagnosed as having intracardiac thrombus and acute pulmonary embolism [7]. Stefani et al. published the case of a 61-year-old woman with BL who presented with a large mass arising from the right ventricle and with associated pericardial and pleural effusions [8]. The mass was almost completely excised but by the time she recovered from the surgery and started systemic treatment the lymphoma was disseminated and she died before completing the first cycle of chemotherapy. Peng et al. presented the case of a 33-year-old man with cardiac BL presenting with a right atrial mass extending into the myocardium and pericardium and with paratracheal lymphadenopathy [9].

In the literature we identified 3 more pediatric cases. Chalabreysse et al. in 2002 described a case of primary BL as part of their review of the only 35 cases of primary cardiac lymphoma published to that date [10]. It was the case of a 9-year-old boy with three intracardiac masses and who was treated successfully with surgical resection followed by multiagent chemotherapy. Fresneau et al. reported the case of a 17-year-old female with cardiac BL arising in the right atrium and with cervical and mediastinal lymphadenopathy [11]. Meshref et al. describe the case of a 10-year-old boy with cardiac BL who presented with multiple intracardiac masses [12]. Finally there are two reported cases of African or endemic BL with primary cardiac involvement [13, 14].

We herein report the case of a 44-year-old woman with primary BL of the heart. This is only the fifth reported case in an immunocompetent adult.

## 2. Case

**Figure 1:**
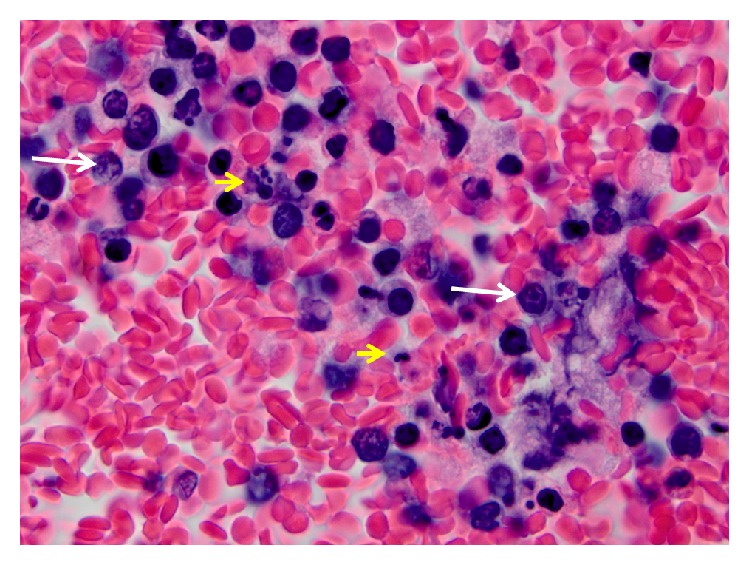
The pericardial fluid was bloody. Within the erythrocytes, numerous atypical intermediate to large cells were seen. Many of the cells had a vesicular chromatin and 1–3 prominent nucleoli (white arrows). Conspicuously, there were numerous apoptotic debris and histiocytes with ingested debris (yellow arrows).

A previously healthy 44-year-old woman presented to the emergency room with early satiety, abdominal bloating, and epigastric discomfort that had started about a week ago. A right upper quadrant ultrasound was done followed by a CT of the abdomen and pelvis. The CT scan showed a partially visualized large pericardial effusion. There was no lymphadenopathy. She then underwent an echocardiogram that showed a hyperdynamic left ventricle systolic function with large circumferential pericardial effusion with tamponade physiology. She was taken to the cath lab where a pericardiocentesis was performed and a total of 730 cc of sanguineous fluid was drained and a pericardial drain was placed. The pericardial fluid contained 65,000 WBCs with the differential showing 6% polys, 1% lymphocytes, 1% monocyte, and 92% highly atypical lymphocytes with moderate basophilic cytoplasm, rare punched-out vacuoles. Most of the large atypical cells had a vesicular nuclear chromatin, some with large nucleolus and marginated chromatin. A hematoxylin and eosin (H&E) stained cell block preparation is shown in Figure 1. This in addition shows numerous apoptotic debris and histiocytes with ingested debris. In tissue sections of BL, the histiocytes and apoptotic debris constitute the starry-sky appearance. Similar findings were seen in this cell block preparation, providing morphological evidence of a highly proliferative lymphoma. Flow cytometry further confirmed the presence of a large B cell population comprising 94% of lymphoid gated events. They were kappa light chain restricted (strong expression of light chains) and were CD19 and CD20 positive. They aberrantly expressed CD10. They did not express CD5 and CD23. FISH was positive for the t(8;14). Overall, the diagnosis was consistent with BL. Diagnosing BL is a challenging process, especially in bodily fluids. However, as shown before [15], the presence of atypical lymphocytes with characteristic morphologic features and confirmatory flow cytometry is very helpful in establishing the diagnosis. FISH for t(8;14) is virtually confirmatory of BL.

**Figure 2:**
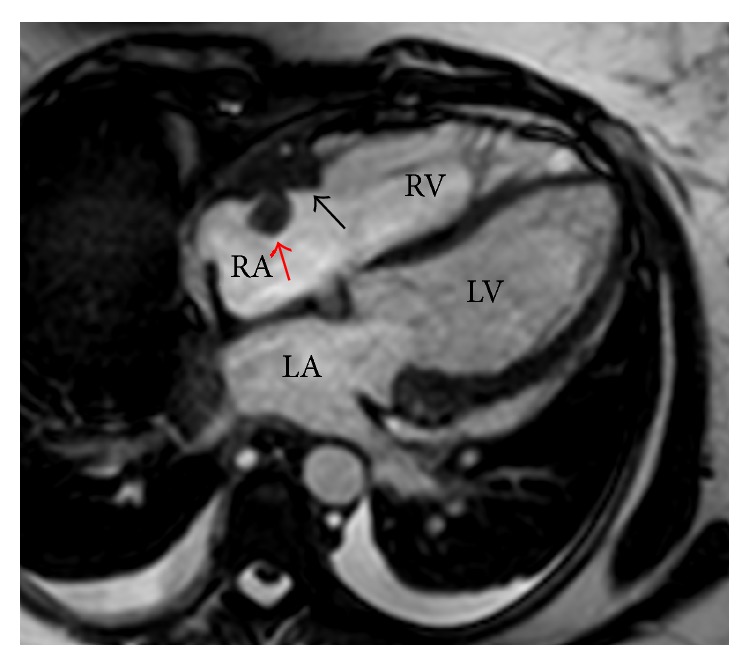
Steady-state free precession magnetic resonance image demonstrating a broad based, isointense structure (black arrow) measuring 3 cm × 1 cm arising from the lateral wall of the right atrium adjacent to the atrioventricular junction of the right atrium. Connected to this broad based mass is an isodense, circular, well-circumscribed mass (red arrow) measuring 1 cm in diameter protruding from the broad based mass into the right atrium. RA: right atrium, RV: right ventricle, LA: left atrium, LV: left ventricle.

For staging she underwent a bone marrow biopsy that was negative for lymphomatous involvement and a CT of the torso that showed a decreased, now small pericardial effusion, stable small bilateral pleural effusions, a 2.6 cm lobulated soft-tissue lesion in the right atrium, and no evidence of lymphadenopathy. A cardiac MRI revealed on T1- and T2-weighted images a broad base, isointense structure measuring 3 cm × 1 cm arising from the lateral wall adjacent to the atrioventricular junction of the right atrium (Figure 2). Connected to this broad based mass, there was an isodense, circular, well-circumscribed mass measuring 1 cm in diameter protruding from the broad based mass into the right atrium. Late gadolinium enhancement images showed heterogeneous enhancement of the broad base mass and no enhancement of the circular mass. Her LDH at presentation was normal and an HIV test was negative. A lumbar puncture revealed no evidence of lymphoma cells in the CSF. The patient was treated with four alternating cycles of modified CODOX-M and IVAC in combination with rituximab [16] and achieved a complete remission. A follow-up cardiac MRI showed no evidence of the right atrial mass. She remains in remission to this date, more than 5 years since her initial presentation.

## 3. Discussion

Primary cardiac lymphomas are extremely rare but unlike most other cardiac tumors fast growing and potentially curable [3, 17–19]. Therefore a high level of suspicion is necessary. Clinical presentation varies and depends on location, size, and growth rate. Dyspnea, lower extremity edema, chest pain, palpitation, and arrhythmia are common presenting symptoms and sometimes might have systemic (or “B”) symptoms such as weight loss, anorexia, fatigue, and night sweats. Heart failure symptoms are due either to intracavity tumor causing obstruction of blood flow through the cardiac chambers or to associated pericardial effusion with cardiac tamponade. BL is faster growing than the other subtypes and patient's presentations might therefore be more acute and dramatic.

Our patient presented with abdominal and epigastric discomfort, which were most likely manifestations of visceral congestion secondary to elevated right heart filling pressures associated with cardiac tamponade.

Certain radiographic characteristics might point towards a lymphoma [20, 21]. The right atrium is the most common site of origin followed by the right ventricle, left ventricle, left atrium, and the interventricular septum. In contrast, the most common type of primary cardiac tumor, myxoma, is most commonly found in the left atrium, generally attached to the fossa ovalis. On CT they appear isoattenuating relative to the myocardium. On CMR they most commonly appear as a large, nodular mass that is isointense or hypointense relative to myocardium on both T1- and T2-weighted images and shows heterogeneous enhancement after administration of gadolinium. Therefore right atrial location, lack of tumour necrosis, an associated pericardial effusion, and infrequent valvular involvement should raise the suspicion for lymphoma. If lymphoma is suspected then a precise pathologic diagnosis should be the next step, as treatment varies for different subtypes. Our case illustrates the usefulness of flow cytometry in the workup of cardiac tumor-associated pericardial effusions immediately after a lymphocytic differential. Cytologic morphology within effusions may be deceiving and does not provide all the necessary information. Therefore clinicians who perform pericardiocenteses in patients with suspected lymphoma of the heart should always order a flow cytometry. Thoracotomy can cause unnecessary treatment delay and should be avoided, especially if diagnosis can be made by less invasive procedures such as percutaneous intracardiac biopsy under ultrasonographic or radiographic guidance, endomyocardial biopsy, or pericardial fluid sampling.

Staging procedures should be similar to the other systemic lymphomas as heart involvement is usually secondary rather than the only location. Infection with HIV needs to be ruled out as part of the initial workup as extranodal involvement in general and heart involvement in particular are more common in HIV related lymphomas [22]. Even though there are not enough data to guide therapy, most patients with cardiac BL have been treated with multiagent chemotherapy with BL's specific protocols. We chose to treat our patient with four alternating cycles of CODOX-M and IVAC rather than three cycles of CODOX-M [23] as we did not feel comfortable calling her low-risk even though she formally met the low-risk criteria used in the LY06 study. We also added rituximab that at least retrospectively is associated with better survival [16]. Our patient received her first cycle of treatment on an EKG monitored bed and did not develop any arrhythmias. Life threatening arrhythmias however have been described after the initiation of chemotherapy and some authors recommend patient monitoring in a cardiac intensive care unit during at least the first cycle [24].

In summary primary cardiac Burkitt lymphoma is an extremely rare and very fast growing tumor of the heart. Suspicion should be high though as cure can be achieved with multiagent chemotherapy.
